# Analysis of Na^+^ concentration patterns in trophectoderm cells of mouse blastocysts using a dual-wavelength electrolyte indicator

**DOI:** 10.1371/journal.pone.0322286

**Published:** 2025-04-30

**Authors:** Ayaka Fujishima, Kazumasa Takahashi, Mayumi Goto, Akiko Fujishima, Takeo Hirakawa, Takuya Iwasawa, Hiromitsu Shirasawa, Yukiyo Kumazawa, Yukihiro Terada

**Affiliations:** Department of Obstetrics and Gynecology, Akita University Graduate School of Medicine, Akita, Japan; University of Tehran, IRAN, ISLAMIC REPUBLIC OF

## Abstract

The developmental process of the mammalian blastocyst involves the intricate interplay of cellular and molecular mechanisms, including electrolyte dynamics within the trophectoderm (TE). We hypothesized that sodium (Na^+^) is actively transported from the TE into the blastocyst cavity, driving water influx and promoting blastocyst expansion. In this study, we investigated the dynamics of Na^+^ concentration in the TE of mouse embryos using sodium-binding benzofuran isophthalate (SBFI), a dual-wavelength Na^+^-sensitive fluorescent indicator. Observations revealed three distinct patterns of Na^+^ dynamics, each correlating with variations in blastocyst cross-sectional area and developmental outcomes. Embryos exhibiting an initial decrease followed by stabilization of Na^+^ concentration (Group A) demonstrated the highest rates of hatching, suggesting a relationship between Na^+^ flux and successful embryonic development. In contrast, embryos with transient increases (Group B) displayed reduced hatching rates and developmental progression. Further, the inhibition of Na^+^/K^+^-ATPase activity disrupted Na^+^ flux and blastocyst cavity expansion, emphasizing its critical role in blastocyst formation. This study highlights the potential of dual-wavelength imaging for elucidating electrolyte dynamics in preimplantation embryos and its implications for optimizing embryo culture systems in reproductive medicine.

## Introduction

In mammalian early embryos, continuous cell division progresses to a distinct developmental stage known as the blastocyst, which is characterized by a unique morphology. The blastocyst is composed of two primary cell types: the inner cell mass (ICM) and the trophectoderm (TE), which encloses a fluid-filled cavity called the blastocyst cavity. As the embryo develops, this cavity expands [1].

In assisted reproductive technologies, embryo culture systems equipped with time-lapse imaging capabilities are becoming increasingly prevalent. Even during the transition from blastocyst formation to the expanded blastocyst stage, human embryos exhibit a range of developmental behaviors [2]. However, the precise developmental significance of these behaviors remains poorly understood. As a result, there is considerable debate regarding the clinical utility of analyzing such behaviors in reproductive medicine [3].

Research on the mechanisms of blastocyst cavity formation and cavity expansion in preimplantation embryos has predominantly focused on mouse models [4,5]. Watson et al. described the formation of gap, tight, and adherens junctions between individual TE cells, which collectively form a sac-like structure [6]. Additionally, water channels, composed of aquaporins, are localized on the apical surface of the TE, while both aquaporins and the Na^+^/K^+^-ATPase pump are present on the basolateral side. These components are believed to regulate the movement of water and electrolytes across the embryo and between cells [5,7,8]. One proposed mechanism suggests that the influx of Na^+^ ions into the blastocyst cavity, mediated by the Na^+^/K^+^-ATPase, generates a concentration gradient, which drives water influx through aquaporins, resulting in the expansion of the blastocyst cavity [8].

The formation of the blastocyst cavity in organisms that fertilize internally and undergo implantation remains poorly understood. However, it can be inferred that during this process, intricate and dynamic exchanges of electrolytes—primarily Na^+^, along with H^+^ and K^+^—occur both within and outside the embryo, as well as between cells. A more refined understanding of electrolyte behavior during embryo development may offer deeper insights into the diverse developmental patterns observed through time-lapse imaging. Furthermore, such knowledge may enhance the development of more physiologically accurate *in vitro* embryo culture systems.

To date, the movement and transport functions of these electrolytes have been inferred indirectly through structural studies and the use of inhibitors [7,8]. However, direct observation of their behavior remains lacking.

In our facility, we recently implemented electrolyte indicators upon embryo development, successfully visualizing and localizing Na^+^ and K^+^ transport within embryos [9]. However, the electrolyte indicator used in prior studies, Corona Green, is prone to leakage from cells [10]. Moreover, single-wavelength indicators are actively expelled by multi-drug resistant proteins, with activity levels varying across cells [11,12].

While current methods, such as single-wavelength electrolyte indicators, have provided valuable insights into electrolyte dynamics, they are not without limitations. For example, single-wavelength indicators can be affected by factors such as variations in dye uptake across different cells, morphological differences between embryos, and the fading of fluorescence over time [13]. These issues may ead to inaccurate estimates of electrolyte concentrations, potentially skewing the interpretation of data. In contrast, dual-wavelength indicators offer the advantage of minimizing these artifacts by providing ratiometric measurements that are less susceptible to fluctuations in dye concentration and cell morphology, thus enhancing the precision and reliability of the data.

In recent years, advancements in dual-wavelength indicators for ion measurement have facilitated the evaluation of intracellular electrolyte concentrations without being affected by cytoplasmic concentration or cell morphology. Notably, reports employing these indicators to measure electrolyte behavior in neuronal and muscle cells have been published [14–17].

**Fig 1:**
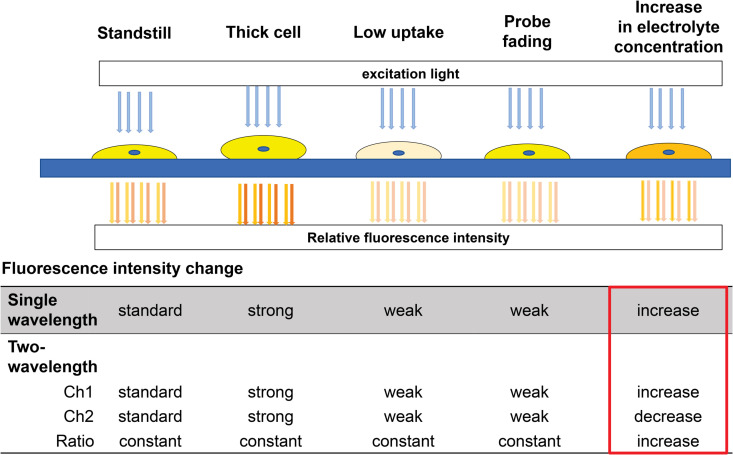
Difference between 1-wavelength and 2-wavelength electrolyte indicators. In 1-wavelength indicators, measurements can be affected by cell thickness, dye uptake, or fading, leading to potential errors. In contrast, 2-wavelength indicators compare two fluorescence channels (Ch1 and Ch2), maintaining a stable ratio when electrolyte concentration is constant. When intracellular electrolyte concentration increases, Ch1 intensity increases while Ch2 decreases, resulting in a higher Ch1/Ch2 ratio (red box).

Sodium-binding benzofuran isophthalate (SBFI) is a widely used Na^+^-sensitive fluorescent indicator that offers sufficient selectivity for Na^+^ with both spatial and temporal resolution, even in the presence of other ions at physiological concentrations. Ratiometric measurements with SBFI can compensate for fluctuating intracellular dye concentrations and are compatible with the same filter equipment used for the Ca^2+^ indicator, Fura-2. When combined with microscopy and ratio imaging, SBFI provides several advantages, including the ability to work with a minimal number of cells, differentiate between intracellular dye and dye that has leaked into the extracellular space, and observe the compartmentalization of the indicator. Upon Na^+^ binding, the fluorescence quantum yield of SBFI increases, the excitation peak narrows, and the excitation maximum shifts to shorter wavelengths, causing a significant change in the fluorescence intensity ratio at 340/380 nm excitation. This enables the relative dynamics of Na^+^ concentrations to be assessed by calculating the fluorescence intensity ratio (340 nm/380 nm) from measurements taken at different excitation and fluorescence wavelengths (Fig 1).

However, there have been no reports on the analysis of electrolyte behavior using dual-wavelength indicators in embryos. Therefore, in this study, we investigated the behavior of Na^+^ concentration in mouse embryos using a dual-wavelength electrolyte indicator. In this study, we investigated the dynamics of Na^+^ concentration in the TE of mouse embryos using sodium-binding benzofuran isophthalate (SBFI), a dual-wavelength Na^+^-sensitive fluorescent indicator. Through this approach, we identified distinct Na^+^ concentration patterns in developing blastocysts and examined their correlation with developmental potential. These findings provide new insights into the role of electrolyte dynamics in preimplantation embryo development and may contribute to optimizing embryo culture conditions in assisted reproductive technologies.

## Materials and methods

### Ethical approval

Approval for the animal studies was obtained from the Institutional Animal Care and Use Committee of Akita University (permission number: a-1–0204). The study adhered to the Animal Experimentation Regulations of Akita University.

### Collection of mouse oocytes, zygotes, and embryos

All animal experiments were conducted in accordance with the Guide for Care and Use of Laboratory Animals of Akita University and were carried out as previously described with minor modifications. Mice were housed in cages at 23°C with a 12-hour light/dark photoperiod and had free access to food and water. *In vitro* fertilization (IVF) was chosen as the embryo acquisition method to ensure precise developmental staging and a uniform environment throughout the experimental period, beginning at insemination. To eliminate strain dependency, female B6D2F1 mice (Charles River Laboratories, Kanagawa, Japan) were chosen because they are an F1 hybrid of C57BL/6 and DBA/2 strains, which minimizes the genetic variability observed in inbred strains, thus reducing strain-dependent effects. A total of five mice, aged 9–14 weeks, were used in this study. Briefly, the experimental animals were intraperitoneally injected with 5 IU of serum gonadotrophin (Serotropin®; ASKA Animal Health Co., Tokyo, Japan), followed by 5 IU of human chorionic gonadotrophin (Gonatropin®; ASKA Animal Health Co.) 48 hours later to induce ovulation. After an additional 14 hours, the females were euthanized by cervical dislocation. Oocytes were collected f and fertilized via IVF with Institute of Cancer Research (ICR) mouse sperm in a human tubal fluid (HTF) medium (Ark Resource, Kumamoto, Japan). Male mice were euthanized by cervical dislocation 1 hour before insemination, and sperm were retrieved from the cauda epididymis by squeezing. The sperm were then suspended in HTF medium at 37°C and 5% CO_2_ for 1 hour and transferred to the IVF medium at a final concentration of 200 sperm/μL. IVF was performed six times in total.

### Embryo culture

Five hours after insemination, IVF embryos were transferred into KSOMaa (Ark Resource) and cultured in 200 μL of the medium in a Primo Vision Embryo Culture Dish (Vitrolife, Gothenburg, Sweden), covered with mineral oil. Embryos were cultured under the following conditions: 5% O_2_, 5% CO_2_, 90% N_2_, and 37°C. The culture medium was not replaced during the experiments. A Well-of-the-Well (WOW) dish system was used for group culture, with each dish containing 200 µL of KSOMaa medium and up to 16 embryos per dish. This system facilitated embryo aggregation, optimizing the culture environment while maintaining an appropriate embryo-to-medium ratio. Embryos were imaged using the Primo Vision time-lapse monitoring system (Vitrolife) at 5-minute intervals from IVF to vitrification, from thawing to experimental use, and from washing after experimental use with the electrolyte indicator for at least 24 hours.

### Vitrification and thawing

Embryos were cultured *in vitro* until they reached the two-cell stage, after which they were vitrified using a Cryotop Safety Kit (Kitazato, Shizuoka, Japan). The two-cell stage was selected for vitrification to maintain consistency with previously established culture systems [9], ensuring comparability between studies. The vitrification period was limited to a maximum of approximately three months. The embryos were immersed in an equilibration solution for 15 minutes and then transferred into a vitrification solution within 90 seconds. Next, they were placed on a Cryotop under a microscope. The Cryotop was frozen directly in liquid nitrogen, with 2–4 embryos vitrified in each Cryotop. For thawing, the embryos were warmed by submerging the Cryotop in thawing solution (1 mol/L sucrose) at 37°C for less than 60 seconds, then transferred into dilution solution (0.5 mol/L sucrose) for 3 minutes. The embryos were immersed in a washing solution twice, for 5 and 1 minute, respectively, washed three times in KSOMaa, and then cultured.

### Observations using the dual-wavelength electrolyte indicator

**Fig 2:**
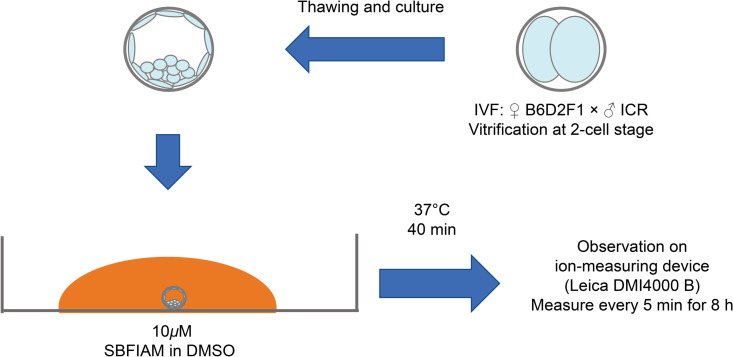
Protocol for acetoxymethyl esters of sodium-binding benzofuran isophthalate (SBFI-AM) and measurement of Na^+^ concentration change using SBFI-AM in mouse blastocysts. Mouse blastocysts, with the blastocyst cavity occupying approximately 40–50% of the total volume (Grade 2), were loaded with 10µM SBFI-AM (in dimethyl sulfoxide) at 37°C for 40 minutes. After loading, imaging was performed every 5 minutes for 8 hours using an ion ratio imaging system (Leica DMI4000 B).

Mouse embryos, with the blastocyst cavity reaching approximately 40–50% of the blastocyst (Grade 2), were treated with 10 µM of acetoxymethyl esters of SBFI (SBFI-AM) (in dimethyl sulfoxide) and incubated at 37 °C for 40 minutes for loading. After the loading period, imaging was performed using an ion ratio imaging system (DMI4000 B; Leica, Nussloch, Germany) (Fig 2). Measurement control was performed with LAS AF ver. 3.0 (Leica). The blastocyst cross-section was focused on the equatorial plane. As an initial setup, the exposure times for the 340 nm and 380 nm excitation lights were set to be the same, and the intensity ratio of 340 nm to 380 nm at the start of the observation was manually adjusted to 1 [18].

**Fig 3:**
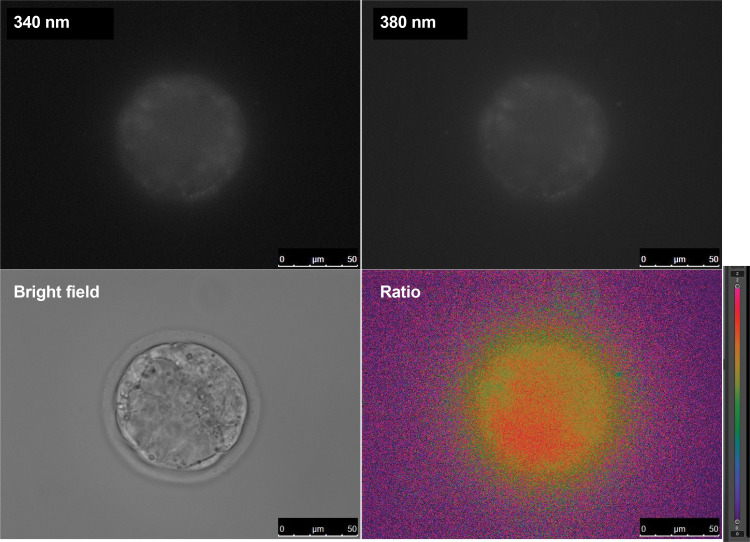
Fluorescence ratio imaging of mouse blastocysts based on 340 nm and 380 nm excitation, with bright-field images. Images were captured for 340 nm and 380 nm excitation, bright-field, and fluorescence intensity ratio. The formula for the fluorescence intensity ratio is: R = 340 nm/380 nm. Ratio images were displayed with color coding in a range from 0 to 2.

Images with 340 nm and 380 nm excitation light, as well as bright-field images, were captured every 5 minutes for 8 hours in the SBFI-AM-added culture medium (Fig 3).

Additionally, observations were conducted under the same conditions with the addition of 1 mM ouabain(Sigma-Aldrich, USA), a specific inhibitor of Na⁺/K⁺-ATPase that blocks active Na⁺ transport across the plasma membrane and disrupts ion homeostasis [19,20], at the time of SBFI-AM loading. Ouabain remained in the culture medium throughout the 8-hour observation period. Untreated embryos served as the control group. After completing the observations with SBFI-AM, the embryos were thoroughly washed and subsequently cultured in KSOMaa. Embryonic development status was evaluated 24 hours later.

### Measurement of Na^+^ ratio and blastocyst cross-sectional area in TE

**Fig 4:**
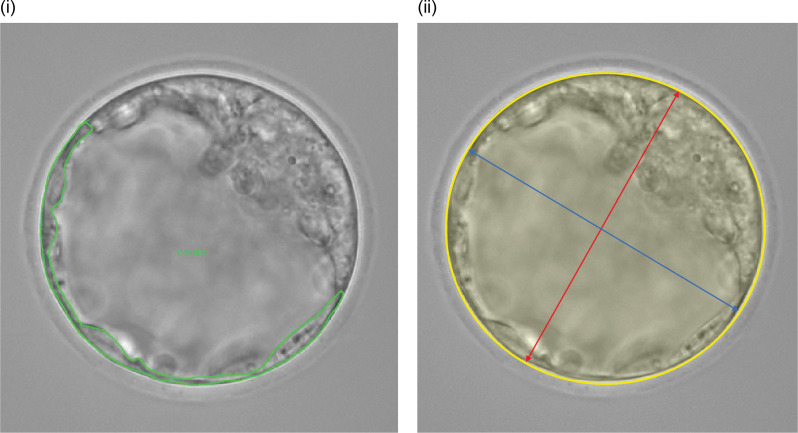
Measurement of TE fluorescence intensity and blastocyst cross-sectional area. The cross-sectional area and trophectoderm (TE) fluorescence intensity were measured every 20 minutes using LAS AF lite. An ROI was manually set to enclose the TE while excluding the ICM, and the fluorescence intensity ratio of SBFI (340 nm/380 nm) was calculated. The blastocyst cross-sectional area (outlined in yellow) was calculated using the following formula: (major axis (red)/2)×(minor axis (blue)/2)×π.

Using LAS AF Lite ver. 4.0 (Leica), the region of interest (ROI) was manually set around the TE, ensuring no overlap with the ICM in 17 mouse blastocysts. Initially, we observed 33 blastocysts and 10 ouabain-treated blastocysts at approximately 40–50% blastocyst cavity expansion. However, embryos with morphological abnormalities or unclear inner cell mass (ICM) structures were excluded from the analysis. As a result, data from 17 blastocysts and 9 ouabain-treated blastocysts were included in the final analysis. Due to the necessity of detailed live imaging analysis for each individual embryo, the number of embryos that could be analyzed was inherently limited. However, all embryos that met the experimental criteria were included to ensure biologically relevant insights. Expanding the ROI to the entire blastocyst would have resulted in overlapping Na+ signals from the TE, ICM, and blastocyst cavity, potentially obscuring their distinct dynamics. To ensure accurate measurement, we focused on the TE region, avoiding overlap with other structures. To accurately define the TE boundary, the ROI was manually traced using bright-field images, where the cellular boundaries are more clearly distinguishable compared to fluorescence images. This approach minimized signal contamination from the blastocyst cavity and ensured that Na+ concentration changes were measured specifically within the TE. The fluorescence intensity ratio of SBFI (340 nm/380 nm) and the blastocyst cross-sectional area were measured every 20 minutes. The cross-sectional area was calculated as (long axis/2) × (short axis/2) × π, as shown in Fig 4. Embryos that hatched during the observation period were measured until hatching. The same measurements were performed for the nine embryos in the ouabain-treated group. After measuring Na+ concentration dynamics and blastocyst cross-sectional area, embryos were classified into three groups (A, B, or C) based on these parameters, with a slight emphasis on Na+ dynamics.

### Statistical analysis

The hatching rates of embryos, categorized based on sodium dynamics patterns, were compared between groups A and B. Embryos were retrospectively classified into Group A, B, or C based on their Na⁺ concentration dynamics and blastocyst expansion patterns. The hatching rate was calculated as the percentage of embryos that had completed hatching 24 hours after the completion of SBFI-AM observation. Statistical analysis was performed using R (R Foundation for Statistical Computing, Vienna, Austria), with inter-group comparisons conducted using Fisher’s exact test. Details on classification criteria are provided in the Results section.

## Results

**Fig 5:**
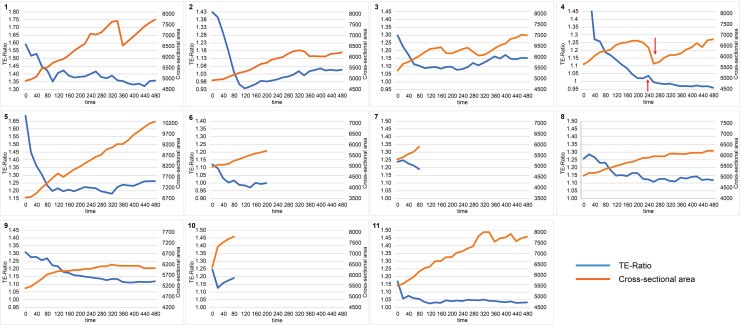
Measurement of Na^+^ ratio in the TE and blastocyst cross-sectional area in Group A. Embryos were classified into three main groups (Groups A–C) according to the time-course changes in Na^+^ concentration and blastocyst cross-sectional area, with Group A considered the most representative. Group A included 11 embryos (Embryo Nos. 1–11) and demonstrated an initial decrease in Na^+^ concentration within the ROI (correspomding to theTE), followed by either a plateau or a gradual increase. The blastocyst cross-sectional area showed consistent growth over time. The differences in the minimum values on the TE ratio axis are attributed to the manual alignment of the starting point for each measurement. In this group, a higher tendency for blastocyst hatching was observed 24 hours after SBFI-AM observation. A transient increase in Na^+^ concentration in the TE just before the collapse in Embryo No. 4 (arrow) was observed.

**Fig 6:**
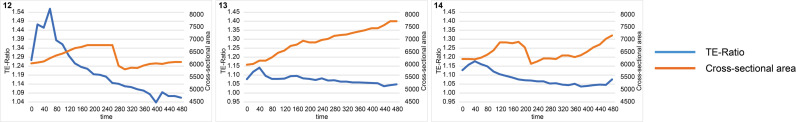
Measurement results of Na^+^ ratio in the TE and blastocyst cross-sectional area in Group B. Embryos were classified into three main groups (Groups A–C) according to the time-course changes in Na^+^ concentration and blastocyst cross-sectional area. Group B included three embryos (Embryo Nos. 12–14) that showed a transient increase in Na^+^ concentration early in the observation period, followed by a decline, while the blastocyst cross-sectional area demonstrated gradual enlargement over time. The differences in the minimum values on the TE ratio axis are attributed to the manual alignment of the measurement start points.

**Fig 7:**
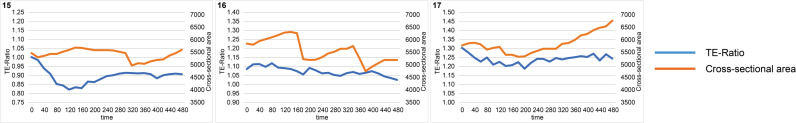
Measurement results of Na^+^ ratio in the TE and blastocyst cross-sectional area in Group C. Embryos were classified into three main groups (Groups A–C) according to the time-course changes in Na^+^ concentration and blastocyst cross-sectional area were classified into three main groups (Groups A–C). Group C included three embryos (Embryo Nos. 15–17) that exhibited patterns distinct from those in Groups A and B. A common feature in Group C was minimal variation in Na^+^ concentration and limited increase in the blastocyst cross-sectional area. The differences in the minimum values on the TE ratio axis are attributed to the manual alignment of the measurement start points.

**Fig 8:**
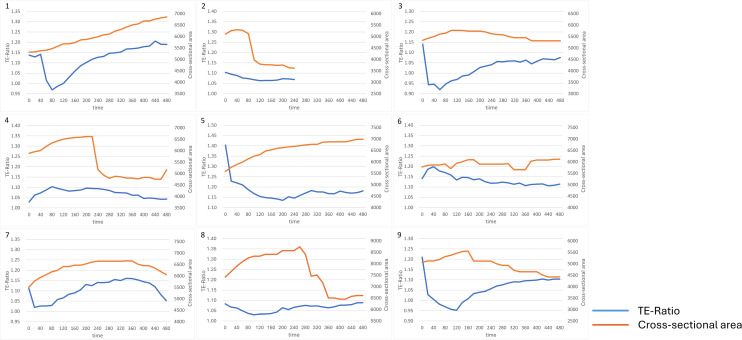
Measurement of Na^+^ ratio in the TE and blastocyst cross-sectional area in the ouabain-treated group. At the time of SBFI-AM loading, 1 mM ouabain was simultaneously added to the culture medium and remained throughout the 8-hour observation period. In the ouabain-treated group, the primary trend observed was a minimal increase in the blastocyst cross-sectional area. The Na^+^ concentration patterns were categorized into two main types: a decrease followed by an increase (Embryo Nos. 1, 3, 7, 9), and no significant change in Na^+^ concentration (Embryo Nos. 2, 4, 6, 8). In the first type, the blastocyst area showed minimal increase, except for Embryo No. 1; in the second type, the blastocysts tended to collapse and decrease in size. Embryo No. 5 exhibited a pattern similar to that of Group A. The minimum values on the TE ratio axis varied due to the manual alignment of the measurement start points.

The measurement results are presented in Figs 5–8. Importantly, Groups A, B, or C were not predetermined experimental conditions. Instead, embryos were retrospectively categorized post-analysis based on the observed Na^+^ concentration dynamics and changes in blastocyst cross-sectional area. All embryos underwent the same experimental procedures. The difference in the minimum values on the TE ratio axis of the graphs was attributed to manual alignment at the start of the measurements. In group A, 11 embryos (numbers 1–11) exhibited a decrease in Na^+^ concentration in the ROI, specifically within the TE, during the early observation phase,as shown in Fig 5. This was followed by either a plateau or a gradual increase in Na^+^ concentration, while the blastocyst cross-sectional area steadily increased over time (Fig 5). In group B, three embryos (numbers 12–14) exhibited a transient increase in Na^+^ concentration during the early observation phase, followed by a decrease in Na^+^ concentration, as shown in Fig 6. The blastocyst cross-sectional area showed a gradual increase over time (Fig 6). Group C, consisting of three embryos (numbers 15–17), displayed patterns that did not fit into either group A or B (Fig 7). A characteristic feature of group C was that the Na^+^ concentration showed minimal changes, and the blastocyst cross-sectional area did not show a marked increase.

The status of the embryos 24 h after the observation with SBFI-AM is shown in Table 1. In group A, five embryos were hatched. Two were in the hatching process, one did not hatch, one was observed to have died, and two remained unconfirmed. In group B, two embryos were in the hatching process, and one did not hatch. In group C, one embryo hatched, and two embryos were in the hatching process.

**Table 1:** Embryo status 24 h after completion of acetoxymethyl esters of sodium-binding benzofuran isophthalate (SBFI-AM) observation.

Embryo No.	Group	Status at 24 hours after completion of observation
1	A	N/A
2	A	Not hatched
3	A	Hatched
4	A	Hatched
5	A	Hatched
6	A	N/A
7	A	Hatched
8	A	Hatching
9	A	Death
10	A	Hatching
11	A	Hatched
12	B	Not hatched
13	B	Hatching
14	B	Hatching
15	C	Hatching
16	C	Hatching
17	C	Hatched

A key trend observed in the ouabain-treated group was the minimal increase in the blastocyst cross-sectional area. The Na^+^ concentration patterns were broadly classified into two types: one in which Na^+^ concentration decreased and then increased (Embryo Nos. 1, 3, 7, and 9) and another in which no change in Na^+^ concentration was observed (Embryo Nos. 2, 4, 6, and 8). In the first pattern, with the exception of embryo 1, the blastocyst cross-sectional area did not increase, whereas embryo 1 showed a gradual increase. In the second pattern, the blastocyst area collapsed and shrank. Embryo 5 exhibited a pattern similar to that of group A (Fig 8).

After the observation in the ouabain-treated group, the developmental status was as follows: two embryos hatched, six were in the hatching process, and one did not hatch (Table 2).

**Table 2:** Embryo status in the ouabain group 24 h after completion of SBFI-AM observation.

Embryo No.	Status at 24 hours after completion of observation
1	Hatched
2	Hatching
3	Hatching
4	Hatching
5	Hatching
6	Hatched
7	Hatching
8	Hatching
9	Not hatched

**Fig 9:**
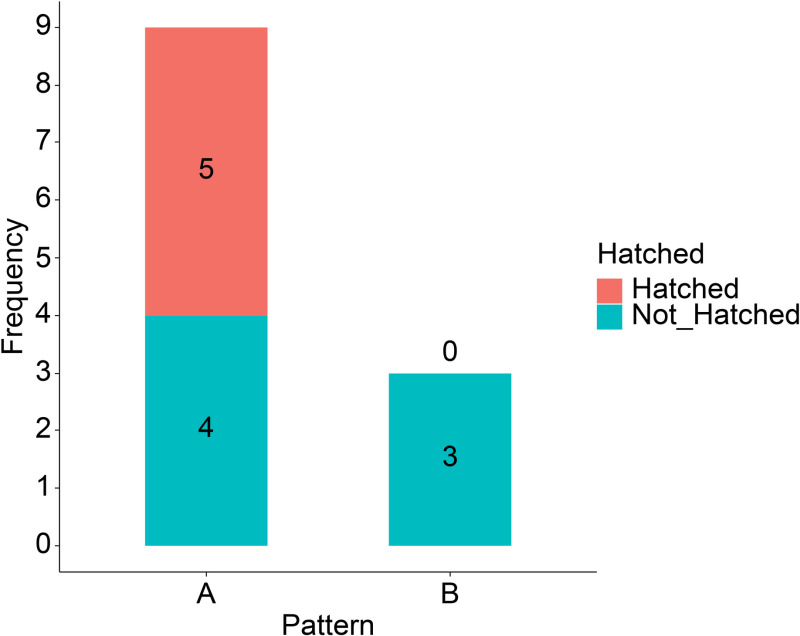
Comparison of hatched blastocyst rates in Groups A and B. The hatching rates of embryos grouped by Na^+^ dynamics patterns were compared between Groups A and B. Statistical analysis was performed using R, and inter-group comparisons were conducted using Fisher’s exact test. The hatching rate of blastocysts tended to be higher in Group A compared to Group B (p = 0.205).

After classifying the embryos and examining the characteristics of each group, we assessed whether differences in developmental potential existed by comparing their hatching rates. This analysis aimed to explore whether differences in Na⁺ concentration dynamics influence developmental potential, as indicated by hatching completion rates 24 hours after SBFI-AM observation. The hatching rate of blastocysts was compared between group A and group B (Fig 9). The hatching rate, defined as the proportion of embryos that had completed hatching 24 hours after SBFI-AM observation, was 70.6% (12/17) in group A and 42.9% (3/7) in group B. The hatching rate tended to be higher in group A compared to group B (p = 0.205).

## Discussion

Numerous researchers have reported the involvement of electrolytes in blastocyst cavity formation [5,7,8,21]. According to Watson et al., gap, tight, and adherence junctions form between TE cells, contributing to the creation of a sac-like structure [6]. Additionally, water channels composed of aquaporins are located on the apical side of TE cells, while electrolyte pumps such as Na^+^/K^+^-ATPase are present on the basolateral side. These structures are thought to regulate the movement of water and electrolytes both within the embryo and across cell boundaries. The influx of Na^+^ ions into the blastocyst cavity via the Na^+^/K^+^-ATPase establishes a concentration gradient, causing water to flow into the blastocyst cavity through aquaporins, ultimately leading to its expansion [8].

Fujishima et al. successfully utilized electrolyte indicators to observe embryo development and directly visualize Na^+^ and K^+^ concentrations [9]. Their study demonstrated that during blastocyst cavity e formation, intracellular Na^+^ concentration decreases while K^+^ concentration increases. In ouabain-treated blastocysts and hatching groups, the inhibition of Na^+^ efflux from cells resulted in an increase in fluorescence intensity. However, in analyses using single-wavelength electrolyte indicators from previous studies, the intracellular indicator concentration might have varied at different developmental stages, or the efflux capacity of the electrolyte indicator could have changed during development. These limitations highlight the need for improved methods to enhance objectivity in future studies.

In this study, we utilized a dual-wavelength electrolyte indicator to observe the samples, minimizing artifacts caused by brightness changes due to sample movement or dye fading. This was achieved by measuring the fluorescence intensity ratio using two distinct excitation and emission settings. If the fluorescence intensities from the two wavelengths are denoted as Ch1 and Ch2, when the electrolyte concentration remains constant, the brightness values of both Ch1 and Ch2 are proportional to the dye concentration within the sample (Fig 1). When the intracellular electrolyte concentration is stable, the ratio of Ch1 to Ch2 also remains constant. This approach eliminates the risk of misinterpreting thick cells as having high electrolyte concentrations, a common issue in single-wavelength measurements. Additionally, it prevents the incorrect interpretation of cells with low dye uptake or those that have faded as having low electrolyte concentrations. When intracellular electrolyte concentration increases, the fluorescence intensity of Ch1 rises, the fluorescence intensity of Ch2 decreases, and the ratio of these wavelengths increases accordingly.

The SBFI-AM used in this study has been previously reported in research investigating Na^+^ transport in mouse renal tubular cells, assessing astrocytic activity, and examining differences in Na^+^/K^+^ pump activity between canine and rat myocardial cells [12–15]. These studies were performed on individual cells, and no prior research has explored these measurements in multicellular structures, such as embryos.

In the present study, we examined the relationship between Na^+^ concentration dynamics in the TE and changes in the blastocyst cross-sectional area. The primary trend observed in Group A suggested that an initial decrease in Na^+^ concentration reflected the efflux of Na^+^ from te TE cells into the blastocyst cavity, which, in turn, triggered water influx into the blastocyst cavity, causing the blastocyst area to increase. Following this, the intracellular Na^+^ concentration appeared to reach equilibrium, while Na^+^ efflux continued into the blastocyst cavity, suggesting ongoing blastocyst expansion. Notably, a transient increase in Na+ concentration in the TE observed just before the collapse in Embryo No. 4 was identified (Fig 5, arrow for Embryo No. 4). This phenomenon likely contributed to the collapse, and it is hypothesized that Na^+^/K^+^ pump inhibition may have occurred prior to collapse. This group demonstrated a tendency for higher hatching rates (hatched) 24 h after observation, suggesting improved embryonic viability.

For Group B, the cause of the initial rise in Na^+^ concentration was unclear. However, the development of the three blastocysts in this group was somewhat inferior compared to Group A, and after the decline in Na^+^ concentration, their patterns became more similar to those of typical groups. The transient increase in sodium observed in these embryos is presumed to have resulted from lower activity of the pumps responsible for sodium efflux into the blastocyst cavity, compared to the influx of sodium into the cells via apical channels.

In Group C, the increase in the blastocyst cross-sectional area was poor, and minimal change in Na^+^ concentration was observed during the observation period.

The addition of ouabain inhibited the Na^+^/K^+^-ATPase, blocking Na^+^ transport from the TE cells to the blastocyst cavity. Consequently, Na^+^ concentration in the TE cells increased while the influx of water into the blastocyst cavity was suppressed, leading to a reduction in the blastocyst cross-sectional area. For Embryo No. 1, while Na^+^ concentration was similar to other ouabain-treated embryos, the blastocyst cross-sectional area continued to increase, though it ultimately failed to complete hatching. This suggests that Na^+^/K^+^-ATPase inhibition interfered with embryo development (hatching).

The rate of blastocyst hatching was higher in group A than in group B, suggesting that embryos in group A may have exhibited more typical development. This could indicate that Na+ concentration dynamics in Group A were more conducive to embryo viability and progression to hatching. However, caution is needed when interpreting these findings due to the limitations of the imaging setup.

The Leica DMI4000 B microscope used for these observations lacked confocal microscopy capabilities, limiting its ability to capture Z-axis data. Due to the spherical shape of the blastocyst, this limitation in imaging depth impacted the accuracy of measurements, particularly when the TE and ICM layers overlapped. Only embryos with a clear separation between the TE and ICM in the equatorial plane were measured, which may not fully reflect Na^+^ dynamics across all regions of the blastocyst. To minimize potential signal contamination, we manually traced the ROI using bright-field images, where cellular boundaries were more clearly distinguishable compared to fluorescence images. This approach allowed us to effectively measure Na⁺ dynamics within the TE while reducing interference from surrounding structures. Future studies using confocal microscopy or advanced imaging techniques could enhance depth and precision.

Furthermore, the dual-wavelength electrolyte indicator used in this study is typically designed for fast signal detection but may require longer observation times for embryos. Despite this challenge, the dual-wavelength system allowed for semi-quantitative analysis of Na^+^ concentration changes over time, providing a valuable method for studying electrolyte dynamics in early embryo development. This approach holds promise for future research, although optimizing the imaging duration and system configuration could improve the accuracy of Na^+^ concentration measurements.

In conclusion, the method developed in this study provides a more objective means of evaluating electrolyte dynamics within embryos, offering valuable insights into Na^+^ transport and its role in blastocyst development. This approach is expected to contribute to the optimization of blastocyst culture media, which could improve embryonic viability in assisted reproduction. Future research should focus on refining this technique to enhance its sensitivity, exploring its application to human embryos, and investigating the impact of various culture conditions on electrolyte balance during early development.
